# Lack of relationship between fasting plasma citrulline and glucose absorption: do not underestimate the role of the failing kidney!

**DOI:** 10.1186/s13054-019-2380-2

**Published:** 2019-04-11

**Authors:** Patrick M. Honore, David De Bels, Andrea Gallerani, Rachid Attou, Willem Boer

**Affiliations:** 1 ICU Department, Centre Hospitalier Universitaire Brugmann-Brugmann University Hospital, Place Van Gehuchtenplein,4, 1020 Brussels, Belgium; 2 Dept. of Anesthesiology, Intensive Care Medicine, Emergency Medicine & Pain Medicine, Ziekenhuis Oost-Limburg, Genk, Belgium

Poole et al. published in a relatively recent paper in *Critical Care* concluding that, while both plasma citrulline concentrations and glucose absorption were reduced in critical illness, fasting plasma citrulline concentrations were not predictive of subsequent glucose absorption and therefore do not appear to be a marker of small intestinal absorptive function [1]. It is argued that the degree of reduction in citrulline concentration may be curtailed by renal dysfunction. Cynober [2] demonstrated that citrulline is mainly metabolized by the kidney, where it is converted into arginine by cells of the proximal tubules [2]. Approximately 83% of the citrulline released by the gut is metabolized within the kidney [2]. Synthesized arginine is released into the general blood circulation. In adults, the citrulline converted by the kidney is enough to provide the body’s full arginine requirements. Arginine synthesized from citrulline represents 60% of the de novo arginine synthesis in the organism [2]. In the Poole study, four patients out of 20 (20%) were in acute kidney injury (AKI) with a creatinine level > 100 μmol/L. These were therefore unable to metabolize a large part of the citrulline released by the gut, hindering interpretation of mean citrulline levels as blunting of the relationship between fasting plasma citrulline concentrations and glucose adsorption is likely. Ware et al. [3] described low levels of citrulline in severe sepsis and a concomitant association with acute respiratory distress syndrome (ARDS). Cynober commented on the Ware publication [4] that, despite the fact that 41% of patients were in AKI, no difference was demonstrated between patients with or without AKI, attributing the absence of expected increase in citrulline levels in AKI to an unknown covariable (for example, gut failure) decreasing citrulline levels [4]. In one study, citrulline (MW = 15 kDa) was reduced by 55% in a session of 90 min of intermittent hemodialysis (IHD) [5]. Continuous renal replacement therapy (CRRT) will likely extract citrulline more efficiently. Of the 41% who were in AKI in the Ware study, half could be expected to be on some forms of renal replacement therapy (RRT), explaining the low citrulline levels in the AKI group. We conclude that patients with AKI or chronic kidney disease (CKD), causing citrulline increase, and patients on RRT, causing a decrease, should be excluded from studies.

## Abbreviations

AKI: Acute kidney injury
ARDS: Acute respiratory distress syndrome
CKD: Chronic kidney disease
CRRT: Continuous renal replacement therapy
IHD: Intermittent hemodialysis
RRT: Renal replacement therapy
